# Transcranial Magneto-Acoustic Stimulation Enhances Cognitive and Working Memory in AD Rats by Regulating Theta-Gamma Oscillation Coupling and Synergistic Activity in the Hippocampal CA3 Region

**DOI:** 10.3390/brainsci15070701

**Published:** 2025-06-29

**Authors:** Jinrui Mi, Shuai Zhang, Xiaochao Lu, Yihao Xu

**Affiliations:** 1 State Key Laboratory of Reliability and Intelligence of Electrical Equipment, Hebei University of Technology, Tianjin 300130, China; 202011402003@stu.hebut.edu.cn (J.M.); 202111402003@stu.hebut.edu.cn (X.L.); 202212901001@stu.hebut.edu.cn (Y.X.); 2 Hebei Key Laboratory of Bioelectromagnetics and Neuroengineering, School of Health Sciences and Biomedical Engineering, Hebei University of Technology, Tianjin 300130, China; 3 Tianjin Key Laboratory of Bioelectromagnetic Technology and Intelligent Health, School of Health Sciences and Biomedical Engineering, Hebei University of Technology, Tianjin 300130, China; 4 School of Health Sciences and Biomedical Engineering, Hebei University of Technology, Tianjin 300130, China

**Keywords:** transcranial magneto-acoustic stimulation, cognitive impairment, phase-amplitude coupling, CA3, rhythm abnormalities

## Abstract

**Background:** Alzheimer’s disease (AD) is a progressive neurodegenerative disorder characterized by cognitive dysfunction and working memory impairment, with early hippocampal damage being a prominent feature. Transcranial magneto-acoustic stimulation (TMAS) has been shown to target specific brain regions for neuroregulation. **Methods:** This study investigated the effects of TMAS on cognitive function, working memory, and hippocampal CA3 neural rhythms in AD rats by specifically stimulating the hippocampal region. **Results**: The novel object recognition test and T-maze test were employed to assess behavioral performance, while time-frequency analyses were conducted to evaluate memory-related activity, neural synchronization, and cross-frequency phase-amplitude coupling. TMAS significantly improved cognitive and working memory deficits in AD rats, enhancing long-term memory performance. Additionally, the abnormal energy levels observed in the θ and γ rhythm power spectra of the CA3 region were markedly restored, suggesting the recovery of normal neural function. This improvement was accompanied by a partial resurgence of neural activity, indicating enhanced inter-neuronal communication. Furthermore, the previously damaged coupling between the θ-fast γ and θ-slow γ rhythms was successfully improved, resulting in a notable enhancement of synchronized activity. **Conclusions:** These findings suggest that TMAS effectively alleviates cognitive and working memory impairments in AD rats and may provide experimental support for developing new treatments for AD.

## 1. Introduction

Alzheimer’s disease (AD) is primarily characterized by progressive cognitive dysfunction and pathological changes in the brain, particularly β-amyloid (Aβ) deposition and neuronal damage [1]. Significant impairments in working and short-term memory have been observed in AD model mice [2]. Abnormal neural oscillations often precede Aβ deposition, notably alterations in θ and γ rhythms associated with learning and memory processes [3]. Intra-hippocampal injection of Aβ oligomers in mice has been shown to induce behavioral and histological changes closely resembling AD pathology, including pronounced spatial memory deficits and a reduction in the peak power of θ and γ oscillations [4].

Compared to single-frequency neural oscillations, the interaction between different frequency bands, known as phase-amplitude coupling (PAC), more effectively reflects the brain’s capacity for neural information integration [5]. PAC, particularly coupling between θ and γ rhythms, is strongly associated with learning and memory functions [6]. Young AD model mice display marked impairments in θ-γ PAC [7]. These pathological alterations emerge during the early stages of AD and coincide with a decline in working memory performance. As the disease progresses, symptoms may advance to mild cognitive impairment and ultimately to clinically diagnosed AD [8,9]. Thus, abnormal neural oscillations have emerged as a promising therapeutic target for AD, underscoring the importance of timely and early intervention in future treatment strategies.

In vivo electrophysiology provides a more comprehensive depiction of neuronal activity in the target region compared to in vitro methods [10,11]. The local field potential (LFP) is an effective tool for measuring electroencephalogram (EEG) signals in specific brain areas. Analysis of EEG signals, particularly the coupling of frequency characteristics across different channels, can serve as a useful biomarker for evaluating spatial memory performance [12]. The complexity of neural activity and functional connectivity revealed by EEG is closely associated with age-related brain dysfunction [13].

Mutual information (MI), a nonlinear analytical approach, has currently gained traction in EEG research for revealing functional connectivity between brain regions. Liu et al. proposed a method for quantifying nonlinear dependencies through MI, emphasizing the frequency-related relationships between time series, and demonstrated that this approach outperforms conventional techniques [14]. By applying MI analysis, it becomes possible to determine the extent to which information from one signal predicts another, accounting for both linear and nonlinear relationships. In this study, MI was calculated between oscillatory frequencies recorded from different channels in the brain, indicating that functional connectivity during working memory tasks may serve as an effective biomarker for detecting working memory impairments in AD.

Transcranial magneto-acoustic stimulation (TMAS) is a novel non-invasive neuromodulation technique that employs static magnetic fields combined with focused ultrasound to stimulate and modulate cranial nerve activity. Norton introduced the potential of using focused ultrasound within static magnetic fields, referred to as TMAS, as an emerging non-invasive brain stimulation method for treating neurological and psychiatric disorders [15,16]. TMAS achieves sub-millimeter spatial resolution even in deep brain structures, offering a focus area nearly ten times greater than that of transcranial magnetic stimulation (TMS) owing to the precision of focused ultrasound delivery [17]. Moreover, TMAS exhibits superior modulation of brain rhythm coupling compared to transcranial ultrasound stimulation (TUS) [18].

Although TMAS has demonstrated cognitive enhancement effects [19], the precise mechanisms underlying its influence on information transmission and memory encoding remain unclear. To further study this issue, TMAS stimulation was first applied to the hippocampus of AD rats before evaluating its therapeutic effects through cognitive motor behavioral tests. To elucidate the potential mechanisms underlying TMAS effects on memory and cognitive function, we analyzed time-frequency characteristics, functional connectivity, and PAC across θ, fast γ, and slow γ frequency. This study investigated the underlying mechanisms by which TMAS modulates neural activity in the hippocampal region of AD model rats.

## 2. Materials and Methods

### 2.1. Animals

The experimental animals were 32 SPF male Wistar rats, 8 weeks of age, weighing 270 ± 20 g. All the experimental rats were purchased from Beijing Huafukang Biotechnology Co., Ltd. (Beijing, China) (License No.: SCXK (jing) 2019-0008). Rats were kept in a 12 h light-dark cycle (temperature 25 ± 2 °C, humidity 50–65%) with free access to diet and drinking water. Sixteen of the 32 rats were randomly selected to establish the AD model. These AD model rats were then randomly assigned to either the AD model group (AD) or the AD stimulation group (TMAS + AD). The remaining 16 normal rats were randomly divided into the normal control group (WT) and the stimulation control group (TMAS + WT). All experimental procedures were reviewed and approved by the Biomedical Ethics Committee of Hebei University of Technology (HEBUTaCUC2022063).

### 2.2. Alzheimer’s Disease Rat Model

The AD group and TMAS + AD group were injected with viral β-amyloid protein (1–42) in the hippocampal region. The WT group and WT + TMAS group were injected with PBS solution. β-amyloid protein (1–42) affects the entire hippocampus and is commonly used to simulate the pathological deposition of Aβ protein observed in AD, making it a standard method for AD model induction. Before the modeling surgery, the β-amyloid protein (1–42) virus needs to be cultured in a water bath for 3 days. Rats were anesthetized using isoflurane at a concentration of 4.0% for induction and maintained at 1.0% during surgery. The rats were positioned in a stereotaxic frame (MSS-35, Stoelting Inc., USA) for accurate injection. The injection site was the dentate gyrus (DG) region of the hippocampus, with coordinates determined from a rat brain atlas (AP: −3.5 mm; ML: ±2 mm; DV: −3.5 mm). A skull drill was used to create holes at the designated locations, and a microsyringe was carefully and vertically inserted into the target brain region to slowly inject 5 μL of virus solution. Following bilateral injections, the scalp was sutured, and the animals were allowed to recover for 3 days.

### 2.3. Microelectrode Array Implantation

The experimental rats were anesthetized prior to surgery and secured in a brain stereotaxic apparatus (51,670, Stoelting Inc., Wood Dale, IL, USA) following complete anesthesia. After removal of the scalp and overlying tissues, a rectangular cranial window measuring 2.0 mm × 0.8 mm was opened above the hippocampal CA3 region (AP: −2.04 mm, ML: −1 mm, DV: −3.8 mm) using an electric cranial drill (68,605, Shanghai Alcott Biotechnology Co., Ltd., Shanghai, China), based on coordinates from a rat brain atlas. An 8-channel microelectrode array (HKP, Plexon Inc., Dallas, TX, USA) was slowly lowered into the dorsal CA3 area of the hippocampus at a rate of 0.02 mm/min using a microelectrode manipulator (MEM, Thomas Recording GmbH Inc., Giessen, Germany) and secured with dental cement. All surgical instruments used on the rats underwent appropriate disinfection procedures throughout the surgery. Postoperatively, the rats were housed for approximately one week with free access to food and water to ensure full recovery.

### 2.4. Transcranial Magneto-Acoustic Stimulation

**Figure 1:**
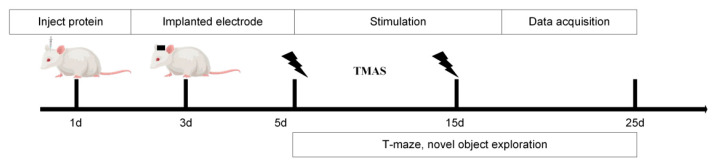
Experimental procedure.

Based on our team’s previous simulation experiments, TMAS demonstrated sufficient intensity to effectively stimulate brain regions even after penetrating the rat skull. The experimental setup is illustrated in Figure 1. Following hair removal to reduce ultrasonic attenuation, the rats were secured in a stereotaxic apparatus. Maintenance anesthesia was provided at 0.5% isoflurane through a facial mask to ensure a stable light anesthetic state.

In this study, static magnetic fields were generated using cylindrical neodymium-iron-boron permanent magnets, each with a diameter of 40 mm and a thickness of 10 mm, positioned bilaterally on either side of the rats’ heads. The magnetic field strength was confirmed pre-experimentally with a Gauss meter. Pulsed ultrasound waves were produced by an arbitrary waveform generator and an RF power amplifier, transmitted through an ultrasound transducer and collimator, and directed through the skull to focus on the hippocampal region. The ultrasound parameters were as follows: a fundamental frequency of 500 kHz, a peak-to-peak voltage amplitude of 0.6 V, 100 cycles per pulse, a pulse repetition frequency of 1.0 kHz, and 100 pulses per sequence. TMAS was applied once daily at 9:00 AM for 2 min over a period of 10 consecutive days. The experimental design included two intervention groups (TMAS + AD and TMAS + WT) that received TMAS treatment and two control groups (AD and WT) that underwent sham stimulation.

### 2.5. Novel Object Recognition

Novel Object Recognition (NOR) is a widely used behavioral assay in animal models to assess changes in memory function [20]. In this study, NOR was employed to evaluate the effects of stimulation on both short-term and long-term memory by setting different retention intervals. The average cognitive index of the WT, TMAS + WT, AD, and TMAS + AD groups was calculated and compared during two NOR tests, with the cognitive index serving as an indicator of learning and memory ability in rats.

Familiarization Phase: Two identical objects (object spheres) were fixed at the bottom of the test box. Each rat was placed into the box, facing away from the objects, and allowed to explore freely for 10 min.

Test 1 (Short-term memory): One hour after the familiarization phase, one of the familiar objects was replaced with a novel object (object cube). Rats were again placed in the box, facing away from the objects, and allowed to explore for 5 min.

Test 2 (Long-term memory): On the following day, one of the remaining familiar objects was replaced with a second novel object (object Mitsubishi column). Rats were reintroduced into the box, facing away from the objects, and allowed to explore for 5 min.

**Figure 2:**
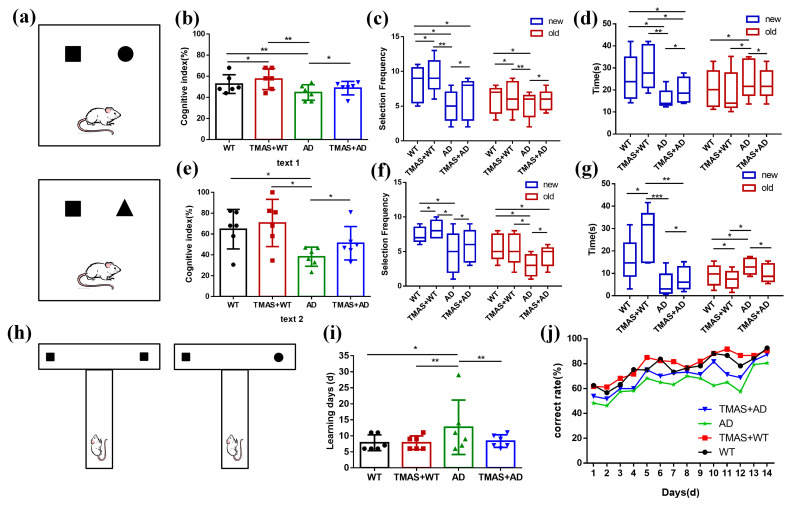
Results of behavioral experiments. (**a**) Schematic diagram of the novel object recognition; (**b**) Cognitive index in Test 1; (**c**) Number of novel objects explored in Test 1; (**d**) Novel object exploration time in Test 1; (**e**) Cognitive index in Test 2; (**f**) Number of new objects explored in Test 2; (**g**) Novel object exploration time in Test 2; (**h**) Schematic diagram of the T-maze; (**i**) Working memory completion time; (**j**) Correct rate for 14 consecutive days. (* *p* < 0.05, ** *p* < 0.01, *** *p* < 0.001).

As shown in Figure 2a, the setup for Test 1 is presented in the upper panel and Test 2 in the lower panel. The square represents the novel object used in Test 1, while the triangle represents the novel object used in Test 2.

### 2.6. T-Maze Test

Rats in the stimulation groups received TMAS treatment for 2 min daily, followed by an 8–10 min rest period before undergoing the T-maze test. Food was restricted for 12 h prior to the T-maze test. Each rat was initially placed at the starting position of the main arm of the T-maze. Upon reaching the choice point, one of the two side arms (left or right) was randomly selected. A food reward had been pre-placed at the end of two arms, and upon locating and consuming the reward, the rat was guided back to the starting position. After a 5 s interval, the rat was allowed to make another choice. If the rat chose the opposite arm on the subsequent trial (i.e., an alternation behavior), it received a food reward again, and this was recorded as a correct choice. Conversely, if the rat selected the same arm consecutively, it was considered an incorrect trial. Each rat performed 20 trials per day, and the daily behavioral accuracy was recorded. The T-maze training was discontinued once a rat achieved an accuracy rate exceeding 80% for two consecutive days. As shown in Figure 2h, squares represent the presence of food, while circles indicate the absence of food.

### 2.7. Local Field Potential

The OmniPlex acquisition system (Plexon, OmniPlex128, USA) was used to record LFPs from the CA3 region of the hippocampus to analyze energy changes and perform time-frequency correlation analyses. Signals were extracted from a 2 s window before and after the memory selection point. Following preprocessing, multi-channel LFP signals underwent time-frequency transformation. A Short-Time Fourier Transform (STFT) was applied to each channel of the multi-channel LFPs to obtain the spatial and temporal distribution of the signal spectrum. This allowed for the investigation of dynamic energy changes in both time and frequency domains during the working memory process. The single-channel LFP signal is denoted as $x\left(t\right)$; its STFT is defined in Equation (1):

(1) $$STFT\left(f,t\right)=\int_{-\infty }^{+\infty }\left[x\left(t\right)g\left(t-\tau \right)\right]e^{-j2\pi ft}dt$$

where $g\left(t\right)$ is the window function. A time window of 2 s with a moving step of 200 ms was used to calculate the dynamic time-frequency distribution of the signals. To evaluate cross-frequency coupling strength, PAC was computed using the instantaneous phase and amplitude of the filtered LFP signals. The Hilbert transform was applied to extract the phase and amplitude corresponding to specific frequency oscillations.

### 2.8. Mutual Information

MI quantifies the degree of dependence between two variables by measuring the extent to which knowledge of one variable reduces the uncertainty of another. This metric captures both linear and nonlinear relationships. In contrast, entropy reflects the inherent uncertainty of a random variable. For a given random variable X, entropy $H\left(X\right)$ represents a fundamental concept in information theory, indicating the uncertainty associated with its possible outcomes. Conditional entropy *H*$\left(X|Y\right)$ denotes the average uncertainty of X given that the value of another random variable Y is known.

(2) $$H=-\underset{x\in X}{\sum }p\left(x\right)\log_{2}p\left(x\right)$$

(3) $$H=-\underset{x\in X,y\in Y}{\sum }p\left(x,y\right)\log_{2}p\left(x|y\right)$$

where $p\left(x\right)$ is the probability density function of *x* and $p\left(x,y\right)$ the joint probability density function of x, y.

(4) $$I\left(X;Y\right)=H\left(X\right)-H\left(X|Y\right)\;=\underset{x\in X,y\in Y}{\sum }p\left(x,y\right)\log_{2}\left(\frac{p\left(x,y\right)}{p\left(x\right)p\left(y\right)}\right)$$

If two variables are independent, their MI is 0. Conversely, if the two variables are identical, the MI reaches 1. A higher MI value indicates a stronger dependence between the variables.

To enable meaningful comparisons across different sampling spaces, it is preferable to use normalized mutual information $I^{*}\left(X;Y\right)\in \left[0,1\right]$. This normalized form allows for a consistent measurement of similarity between variables in various experiments. The normalization process eliminates the influence of variability in the estimation of $H\left(X\right)$ on the calculation of the mutual information matrix distance. The normalized mutual information is defined as follows:

(5) $$I^{*}\left(X;Y\right)=\frac{I\left(X;Y\right)}{min\left(H\left(X\right),H\left(Y\right)\right)}$$

In practice, directly estimating the probability distribution of variables can be challenging, especially in high-dimensional spaces with limited data samples, as is often encountered in neural recordings. The calculation of MI is particularly sensitive to statistical errors when the number of sampling points is insufficient. For instance, in cases involving short recordings with *k* sampling points (*k* < 50), the estimation of MI may exhibit substantial variance, whereas this sample size is generally adequate for computing correlation coefficients.

### 2.9. Statistical Analysis

Data are presented as mean ± standard deviation (SD). Behavioral data were analyzed using one-way repeated-measures analysis of variance (ANOVA). One-way ANOVA and least significant difference (LSD) post hoc tests were employed to assess differences between groups. All statistical analyses were conducted using GraphPad Prism 8 (GraphPad Software Inc., San Diego, CA, USA). A *p*-value < 0.05 was considered statistically significant.

## 3. Results

### 3.1. TMAS Improved the Learning Memory Ability of AD Rats

In the novel object recognition test, the results of Test 1 and Test 2 are shown in Figure 2b–g and Table 1 and Table 2. During Test 1, the cognitive index of rats in the AD group was significantly lower than that of the WT group. Significant differences were also observed between the AD and TMAS + AD groups, as well as between the WT and TMAS + WT groups (*p* < 0.05). The AD group exhibited significantly reduced exploratory frequency and duration toward the novel object compared to the WT group (*p* < 0.05). TMAS significantly enhanced novel object exploration in AD model rats, evidenced by increased contact frequency (*p* < 0.05) and extended investigation duration (*p* < 0.05). Test 2 exhibited superior cognitive enhancement relative to Test 1. In Test 2, the cognitive index of rats in the TMAS + AD group was significantly higher than that of the AD group (*p* < 0.05). Both TMAS-treated groups (TMAS + WT and TMAS + AD) exhibited significantly increased exploratory preference for novel objects post-stimulation (*p* < 0.05). These findings suggest that TMAS enhances both short-term and long-term memory function. This enhancement was particularly pronounced for long-term memory consolidation.

### 3.2. TMAS Improved the Working Memory Function of AD Rats

The T-maze is a widely used behavioral method for assessing working memory [21]. The results of the T-maze are shown in Figure 2i,j and Table 3. As shown in Figure 2i, the AD group required an average of 37.33% more days to reach the learning criterion compared to the WT group, indicating significant working memory impairment. In contrast, normal rats subjected to TMAS exhibited a 13.33% reduction in learning duration, suggesting improved working memory performance. Similarly, TMAS significantly shortened the learning period in the TMAS + AD group (*p* < 0.05), though a gap remained between the TMAS + AD and WT groups. Compared with the WT group, the AD group consistently exhibited lower accuracy. The TMAS + AD group demonstrated a marked improvement in accuracy following stimulation (*p* < 0.05). Overall, the T-maze results indicate that the AD group suffered from substantial working memory deficits compared to the WT group. Although TMAS did not fully reverse these impairments, it significantly improved the learning and memory capabilities in both normal and AD rats, enabling them to complete the working memory task more efficiently.

### 3.3. TMAS Improved Abnormal Local Field Potential in AD Rats

**Figure 3:**
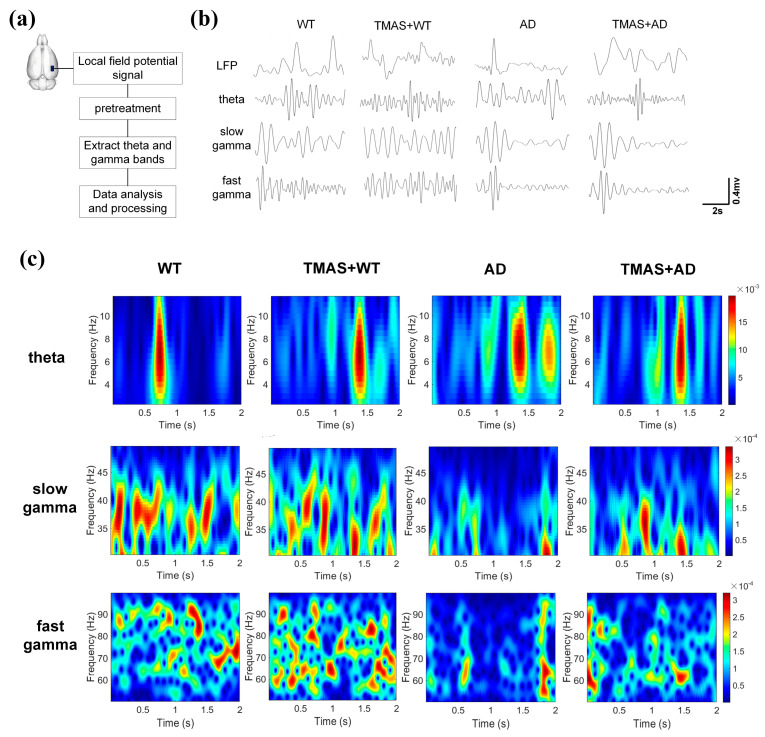
(**a**) Experimental flow chart; (**b**) local field potential signal after pretreatment; (**c**) energy distribution of time−frequency signals.

This study investigated the effects of TMAS on rhythmic oscillations related to working memory in rats during the T-maze test. As shown in Figure 3b, the pre-treatment LFP signals were captured, and Figure 3c presents the corresponding time–frequency energy distribution of θ, slow γ, and fast γ oscillations during the same period. The horizontal axis represents time, the vertical axis denotes frequency, and color depth reflects energy levels (yellow indicating high energy, blue indicating low energy). The selection moment is at the 1 s mark. θ power was significantly reduced in the AD group compared to the WT group. TMAS stimulation enhanced neuronal activity in the TMAS + AD and TMAS + WT groups. Slow γ oscillations (30–50 Hz) demonstrated preferential power distribution below 40 Hz. The AD group exhibited generally weak neural excitability within the 2 s window, whereas TMAS stimulation markedly increased neuronal excitability in the TMAS + AD group. In the fast γ range, the WT and TMAS + WT groups showed a broad and dispersed energy distribution, reflecting higher neuronal activity. TMAS intervention increased fast γ energy in the TMAS + AD group. Overall, slow and fast γ oscillation energy levels in AD rats were significantly lower than those in normal Wistar rats. TMAS increased these energy levels in both normal and AD rats, indicating that TMAS can effectively restore abnormal power spectrum energy and enhance neuronal excitability in AD rats, especially in γ oscillations.

### 3.4. TMAS Enhanced Synergy in the CA3 Brain Region

**Figure 4:**
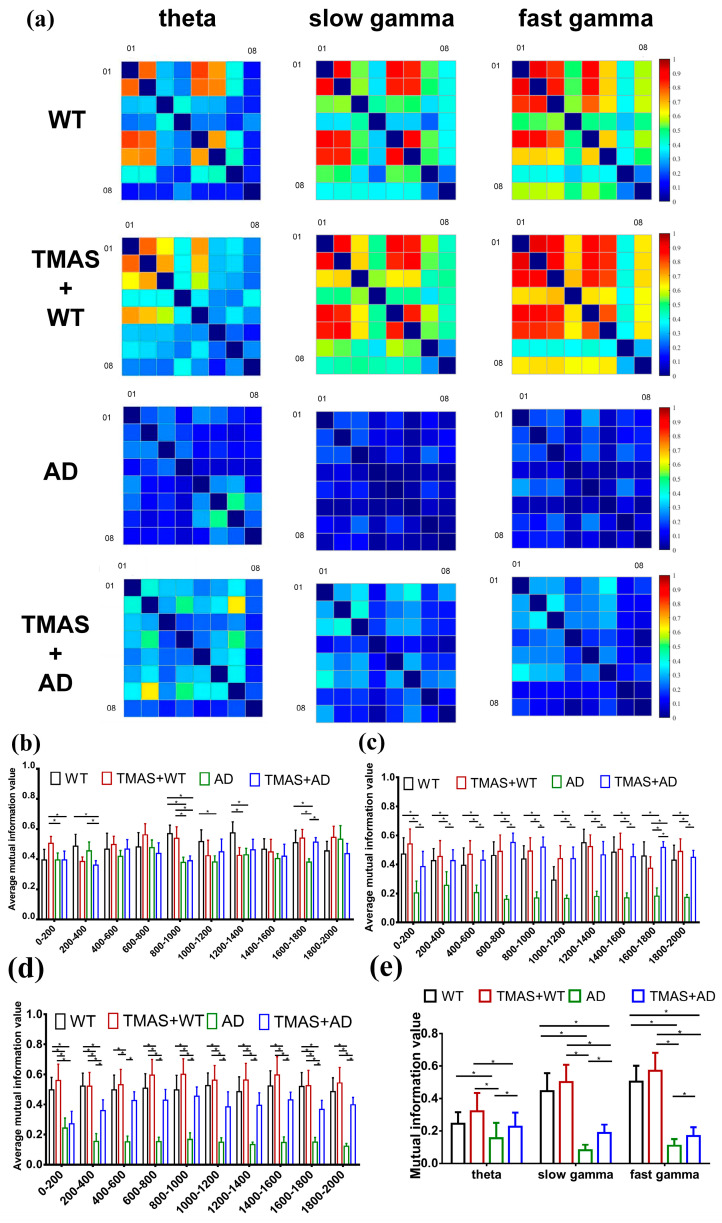
Results of mutual information analysis. (**a**) A series of mutual information matrices of electrophysiology recordings at different oscillations: theta, slow gamma, fast gamma; (**b**) theta mutual information in steps of 200 ms; (**c**) slow gamma mutual information in steps of 200 ms; (**d**) fast gamma mutual information in steps of 200 ms; (**e**) average mutual information value. (* *p* < 0.05).

In this study, MI analysis was employed to assess neuronal synchrony in the hippocampal CA3 region during working memory tasks. The results of the MI are shown in Figure 4 and Table 4 and Table 5. Figure 4a presents the computed MI matrices for all experimental groups. The x- and y-axes represent the eight recording channels, and each matrix cell reflects the MI value between channel pairs; diagonal values are set to zero. Lighter shades indicate higher MI values, representing stronger rhythmic synchronization and greater cooperation among neuronal clusters, whereas darker shades indicate lower MI and weaker synchrony.

The MI matrices revealed that θ, slow γ, and fast γ oscillations in the AD group displayed poor neuronal synchrony. Following TMAS stimulation, MI values for the TMAS + AD groups increased significantly in the theta band compared to the AD group (*p* < 0.05). The average MI value for slow γ oscillations in the AD group was 18.10% of that in the WT group. The MI value in the TMAS + AD group increased by 22.77% relative to the AD group. Similar trends were observed in the fast γ spectrum. Overall, TMAS significantly elevated MI values in the LFP signals from the CA3 region, especially in the γ oscillation bands, and moderately increased MI in the theta band. These findings suggest that TMAS enhances neuronal synchrony and oscillatory activity in the hippocampal CA3 region.

The preliminary MI matrix suggested that TMAS enhances the rhythmic oscillations of neuronal clusters. To further illustrate these changes, the data from each channel were segmented into 10 intervals with a 200 ms step size. The MI values from these segments were calculated and averaged to analyze the temporal dynamics of θ, slow γ, and fast γ rhythms in each group separately (Figure 4b,d). TMAS significantly influenced θ oscillatory activity during the 800–1400 ms interval, corresponding to the period of working memory selection. During the 800–1400 ms interval, the MI values of slow γ and fast γ oscillations in the AD group remained significantly lower than those in the WT group (*p* < 0.05). These findings indicate that TMAS stimulation significantly increased MI values in the TMAS + AD group compared to the AD group (*p* < 0.05), demonstrating that TMAS effectively enhances neuronal synchrony and oscillatory activity in the CA3 region.

### 3.5. Modulating Effect of TMAS on Theta–Gamma Phase–Amplitude Coupling

**Figure 5:**
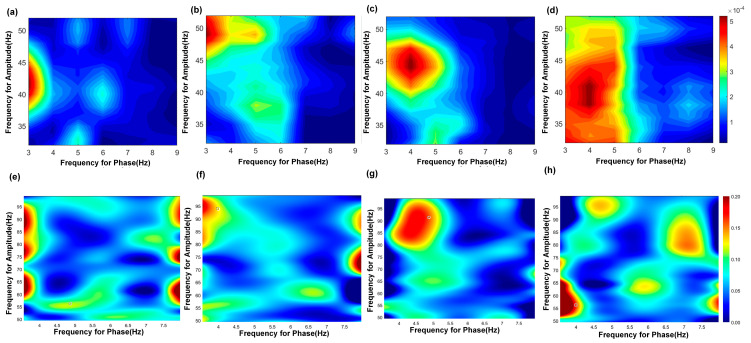
PAC results of theta−gamma in the CA3 region. (**a**–**d**) show the PAC results of theta−slow gamma; (**a**) AD group, (**b**) TMAS + AD group, (**c**) WT group, (**d**) TMAS + WT group. (**e**–**h**) show the PAC results of theta−fast gamma; (**e**) AD group, (**f**) TMAS + AD group, (**g**) WT group, (**h**) TMAS + WT group.

The results of the PAC are shown in Figure 5 and Table 6. PAC quantifies the interaction between the phase of low-frequency oscillations and the amplitude of high-frequency rhythms, reflecting physiological mechanisms whereby the phase of slow oscillations governs local neuronal excitability, and increases in high-frequency amplitude indicate elevated population synaptic activity or selective activation of cortical subnetworks. In the hippocampal CA3, θ phase (3–6 Hz) preferentially modulated slow γ amplitude (30–50 Hz) during exploration. TMAS restored the modulation index to (4.14 ± 1.00) × 10^−4^ (vs. AD: *p* < 0.05), correlating with θ–slow γ PAC coherence increase. Overall, the abnormal neural oscillatory activity in the CA3 region of AD rats during working memory tasks is characterized by reduced coupling of both θ–slow γ and θ–fast γ oscillations. TMAS appears to alleviate these deficits by restoring PAC strength and improving the coordination of these oscillatory patterns.

## 4. Discussion

TMAS enables deep brain stimulation with exceptional focusing precision. In 2003, Norton first proposed the potential of using ultrasound to generate electric fields within magnetic fields for neuromodulation [15]. Subsequent studies confirmed that TMAS technology can effectively modulate neural electrical activity [22]. In our previous work, we demonstrated that TMAS can generate electric fields in conductive media, oriented perpendicular to both the magnetic and acoustic fields, with the electric field distribution closely matching that of the acoustic field. In this study, TMAS exhibited beneficial effects on theta and gamma rhythms in the CA3 region of AD model rats. Unlike TMS, which typically uses repetitive magnetic stimulation at specific frequencies, TMAS applies a static magnetic field. Prior studies indicated that high-intensity static magnetic fields (1.5 T) and prolonged exposure (3 h) could influence biological samples, such as altering Na^+^ and Ca^2+^ levels in rat brains [23]. To minimize potential tissue damage, we employed a low-intensity magnetic field (0.3 T) and short exposure times (60 s) in this study.

The NOR and T-maze tests are widely used to assess spatial and working memory in rodents. Numerous studies have demonstrated that AD model rats exhibit cognitive and working memory impairments. Mehak et al. reported deficits in working memory linked to abnormal neural excitability in key brain regions, including the hippocampus [24]. In our experiment, AD was induced by injecting Aβ protein into the dorsal hippocampus, and behavioral tests confirmed significant impairments in both cognitive and working memory abilities. These results align with previous findings in the literature. Notably, TMAS significantly improved the performance of AD rats in both the NOR and T-maze tests, indicating a restoration of spatial and working memory. In the present in vivo behavioral study, TMAS treatments showed therapeutic efficacy in the AD model.

The activity of hippocampal place cells is crucial for spatial learning and memory. In AD, the instability of place cell activity can lead to errors in spatial information processing and impairments in spatial memory function [25]. The θ and γ rhythms in the CA3 region are essential for cognitive and memory processes, with γ oscillations likely contributing to the coordination of place cell activity during spatial memory tasks. Moreover, the coupling between θ and γ rhythms reflects dynamic information processing mechanisms [26]. Studies in AD models have shown reduced power in θ, low-γ, and high-γ oscillations, suggesting a state of low neuronal excitability that compromises the neural processes underlying spatial learning [27,28]. Recent research on hippocampal function has consistently demonstrated that the power spectral densities of θ and γ oscillations in AD rats are significantly diminished. These alterations are accompanied by reduced oscillatory activity and abnormal neuronal excitability, which appear earlier than overt behavioral dysfunction [29]. In addition, AD is associated with decreased θ–γ phase–amplitude coupling, consistent with our findings of disrupted hippocampal rhythms in AD model rats. LFP spectral analysis revealed significantly decreased power spectral density for θ, slow γ, and fast γ oscillations in the CA3 region of AD rats during working memory performance. The θ oscillatory energy in AD rats appeared dispersed and was notably weaker in the 0–0.5 s window before the decision point compared to WT rats. In contrast, γ oscillation energy was reduced throughout the entire 0–2 s window. Importantly, TMAS treatment ameliorated these abnormal power spectra in the hippocampal CA3 region of AD rats. Similarly, stimulated WT rats showed increases in both θ and γ oscillatory energy compared to their non-stimulated counterparts. Although TMAS maintained its beneficial effect on θ oscillations during working memory, its impact on abnormal θ oscillations in AD rats was less marked. However, TMAS significantly elevated both slow and fast γ energy levels in AD rats, suggesting that the observed improvements in working memory performance may primarily result from the modulation of γ oscillatory activity in the hippocampus.

MI, derived from information entropy, reflects the non-stationary characteristics of EEG signals and represents the activity and coordination of neuronal clusters. Since both EEG and LFP signals capture neural activity, information entropy can similarly describe the non-stationarity of LFPs. In this study, MI analysis across eight channels revealed a significant decrease in AD rats, indicating impaired neural oscillations in the θ, slow γ, and fast γ frequency bands. TMAS notably enhanced neuronal oscillatory activity in the CA3 region, particularly in γ oscillations. Hippocampal volume atrophy, neuronal degeneration, and reduced neural activity are well-documented features of AD pathology [30]. By improving neuronal activity within the hippocampus, it may be possible to mitigate the progression of AD and achieve therapeutic benefits [31]. The study findings suggest that TMAS enhances neuronal firing rates and coordination in the CA3 region, potentially serving as a mechanism by which TMAS alleviates cognitive decline and working memory deficits associated with AD.

θ and γ oscillations interact via cross-frequency PAC, where the phase of low-frequency oscillations modulates the amplitude of high-frequency rhythms. This mechanism is prevalent in brain regions involved in cognitive processing [32]. PAC can be assessed by recording hippocampal network activity, and studies have reported reductions in θ-high γ coupling in APP mutant mice at various ages [33]. The hippocampal CA1 and CA3 regions are both crucial for working memory and cognitive functions; however, most research has focused on θ–γ PAC in CA1, with limited attention to CA3. Previous studies indicate that impaired θ–γ PAC is evident in patients with AD-related dementia and worsens as the disease progresses [34]. In our study, we observed disrupted θ–slow γ and θ–fast γ PAC in both the CA1 and CA3 regions of AD rats. The results demonstrate reduced coordination of neuronal activity and abnormal oscillatory patterns in the CA3 region of AD rats, leading to impaired θ–slow γ and θ–fast γ PAC. This disruption may contribute to the early deficits in memory processing observed in AD. Notably, continuous TMAS stimulation partially restored θ–slow γ and θ–fast γ coupling in AD rats, underscoring the importance of θ–γ PAC as a key factor in cognitive aging and memory decline. TMAS appears to improve these impairments by enhancing oscillatory synchronization and neural connectivity in the hippocampus.

In previous studies, researchers have conducted a series of experiments to investigate the effects of TMAS on the molecular level in the brain tissue of AD mice. One study showed that TMAS alleviates AD pathology and improves cognitive and memory functions [35]. In addition, researchers found that TMAS treatment stimulated microglial proliferation and migration while enhancing the phagocytosis and clearance of Aβ [36]. In our study, we focused on the modulation of neural oscillations in the CA3 region of AD rats and examined the therapeutic effects of ultrasound from the perspective of neural oscillations. Unlike previous studies, our approach provides a different perspective on the therapeutic effects of TMAS in AD rats. However, a limitation of our study is that it is more macroscopic in nature and therefore lacks detailed exploration of the molecular mechanisms. For example, we do not yet know whether or how TMAS alters neural oscillations through molecular changes in brain tissue. In future research, we will address these questions in greater depth.

Ultrasound-mediated activation of Piezo channels (e.g., Piezo1/2) enhances neuronal excitability via mechanotransduction, as established in prior studies [37]. Potassium, sodium, and calcium voltage-gated channels have also been identified as targets [38,39], contributing to TMAS-induced neuronal action potentials. These excitability changes likely alter LFP coding and spike–LFP coupling, establishing new neural oscillation patterns. Future research will use optogenetic or chemogenetic approaches to verify the ion channel mechanisms involved in TMAS regulation of CA3 oscillations.

Currently, TMS represents a commonly used noninvasive physical therapeutic technique for Alzheimer’s disease (AD) in clinical settings [40]. Research has shown that 20 Hz and 1 Hz rTMS could improve cognitive impairment in AD mice [41]. Additionally, previous research has shown that TMS can affect cellular redox status and the amyloid production process, increase the excitability of the cerebral cortex, promote synaptic plasticity, and enhance the cognitive abilities of AD [42]. However, due to the limited penetration depth of magnetic fields, TMS cannot effectively stimulate deep brain regions, such as the hippocampus, and is incompatible with MRI for precise navigation [43]. In comparison, TMAS offers higher spatial resolution and stimulation depth and can precisely target different deep brain regions. Furthermore, prior studies have established that TMAS demonstrates superior neuromodulatory efficacy compared to TUS [44]. We believe that TMAS has the potential to become a new physical modulation tool for the clinical treatment of AD.

Overall, this study demonstrates that TMAS targeting the hippocampal CA3 region effectively modulates θ and γ oscillations in AD rats. These findings suggest that TMAS holds promise as a therapeutic strategy for AD by enhancing both long-term learning and working memory, alongside improving neuronal excitability and synchronization. However, this research was limited to oscillatory activity within the CA3 region; future studies should extend the investigation to other hippocampal subregions and additional brain areas involved in memory networks. As neural oscillations result from the interplay between excitatory and inhibitory neuronal populations, the specific modulatory effects of TMAS on these neuronal circuits in the context of AD remain to be elucidated. Furthermore, only a single set of stimulation parameters was employed in this study; subsequent research should explore a range of TMAS parameters to optimize its therapeutic efficacy.

## 5. Conclusions

This study investigated the power spectrum and correlation of θ, slow γ and fast γ oscillations in LFP signals from WT, TMAS + WT, AD, and TMAS + AD groups, alongside behavioral performance differences assessed by the NOR and T-maze tests. Results showed that θ power increased while γ power decreased in AD rats. Behavioral assessments indicated that TMAS significantly improved cognitive and working memory, with notable enhancements in long-term memory as demonstrated in the NOR test. Moreover, LFP analysis revealed that TMAS markedly elevated γ oscillation power in AD rats, particularly in the fast γ range, and this enhancement persisted over time. Although the present study focused on the short-term effects of TMAS stimulation, future research should explore its long-term therapeutic potential in AD models. An AD rat model exhibiting hallmark pathological features was successfully established, and TMAS intervention was shown to effectively enhance learning and cognitive functions by targeting the hippocampus. Notably, TMAS significantly increased γ oscillatory activity in the CA3 region, a key area implicated in memory processes. Collectively, these findings provide experimental support for the potential application of TMAS as a therapeutic strategy for AD.

## Figures and Tables

**Table 1:** The results of Test 1 of the NOR.

		WT	TMAS + WT	AD	TMAS + AD
cognitive index (%)		52.59 ± 3.63	57.36 ± 4.06	44.59 ± 3.02	48.78 ± 2.60
average selection frequency	old	6.00 ± 0.89	6.40 ± 1.02	5.80 ± 1.15	6.40 ± 0.74
	new	8.20 ± 1.35	8.60 ± 1.43	6.40 ± 1.91	7.00 ± 1.89
average selection time (s)	old	20.75 ± 4.34	19.88 ± 5.49	22.73 ± 5.63	23.11 ± 4.01
	new	25.22 ± 4.77	25.58 ± 6.14	21.75 ± 8.64	22.25 ± 6.13

**Table 2:** The results of Test 2 of the NOR.

		WT	TMAS + WT	AD	TMAS + AD
cognitive index (%)		65.58 ± 11.70	61.78 ± 11.31	38.25 ± 3.74	47.68 ± 8.03
average selection frequency	old	5.60 ± 0.87	5.40 ± 1.02	4.00 ± 0.89	4.40 ± 0.67
	new	7.40 ± 0.51	8.20 ± 0.58	4.80 ± 1.35	5.80 ± 1.06
average selection time (s)	old	16.15 ± 5.88	16.15 ± 5.88	10.01 ± 7.67	11.74 ± 9.64
	new	10.75 ± 1.33	10.01 ± 1.57	8.21 ± 2.77	7.02 ± 3.24

**Table 3:** The results of the T-maze.

	WT	TMAS + WT	AD	TMAS + AD
Days (d)	7.50 ± 1.19	6.50 ± 0.28	10.30 ± 2.23	8.66 ± 1.45
average accuracy rate (%)	76.77 ± 2.76	79.62 ± 2.72	63.59 ± 2.63	69.94 ± 2.81

**Table 4:** MI in steps of 200 ms.

		WT	TMAS + WT	AD	TMAS + AD
0–200 s	θ	0.39 ± 0.07	0.46 ± 0.04	0.36 ± 0.05	0.36 ± 0.06
	slow γ	0.47 ± 0.11	0.49 ± 0.10	0.19 ± 0.08	0.35 ± 0.11
	fast γ	0.50 ± 0.08	0.51 ± 0.11	0.23 ± 0.07	0.25 ± 0.08
200–400 s	θ	0.49 ± 0.08	0.35 ± 0.03	0.41 ± 0.06	0.33 ± 0.03
	slow γ	0.43 ± 0.08	0.41 ± 0.11	0.24 ± 0.09	0.39 ± 0.08
	fast γ	0.52 ± 0.09	0.47 ± 0.09	0.15 ± 0.05	0.32 ± 0.07
400–600 s	θ	0.47 ± 0.10	0.45 ± 0.05	0.38 ± 0.04	0.42 ± 0.06
	slow γ	0.39 ± 0.12	0.43 ± 0.09	0.19 ± 0.05	0.39 ± 0.07
	fast γ	0.50 ± 0.10	0.48 ± 0.10	0.15 ± 0.04	0.38 ± 0.06
600–800 s	θ	0.48 ± 0.09	0.51 ± 0.07	0.43 ± 0.05	0.40 ± 0.07
	slow γ	0.46 ± 0.09	0.45 ± 0.11	0.15 ± 0.03	0.49 ± 0.07
	fast γ	0.51 ± 0.10	0.54 ± 0.10	0.15 ± 0.03	0.38 ± 0.07
800–1000 s	θ	0.57 ± 0.06	0.48 ± 0.08	0.35 ± 0.03	0.35 ± 0.03
	slow γ	0.44 ± 0.09	0.45 ± 0.09	0.16 ± 0.04	0.47 ± 0.07
	fast γ	0.50 ± 0.10	0.54 ± 0.10	0.16 ± 0.04	0.41 ± 0.06
1000–1200 s	θ	0.52 ± 0.08	0.39 ± 0.10	0.35 ± 0.04	0.40 ± 0.08
	slow γ	0.29 ± 0.09	0.40 ± 0.09	0.16 ± 0.02	0.39 ± 0.08
	fast γ	0.52 ± 0.08	0.51 ± 0.10	0.14 ± 0.03	0.34 ± 0.10
1200–1400 s	θ	0.58 ± 0.07	0.39 ± 0.05	0.39 ± 0.04	0.42 ± 0.07
	slow γ	0.55 ± 0.09	0.48 ± 0.08	0.17 ± 0.04	0.42 ± 0.09
	fast γ	0.49 ± 0.10	0.51 ± 0.11	0.13 ± 0.02	0.35 ± 0.08
1400–1600 s	θ	0.47 ± 0.07	0.41 ± 0.08	0.37 ± 0.03	0.38 ± 0.08
	slow γ	0.49 ± 0.10	0.46 ± 0.11	0.16 ± 0.04	0.41 ± 0.09
	fast γ	0.52 ± 0.10	0.54 ± 0.12	0.15 ± 0.04	0.39 ± 0.05
1600–1800 s	θ	0.51 ± 0.08	0.49 ± 0.06	0.34 ± 0.02	0.46 ± 0.03
	slow γ	0.46 ± 0.10	0.34 ± 0.08	0.17 ± 0.06	0.47 ± 0.04
	fast γ	0.52 ± 0.09	0.48 ± 0.10	0.15 ± 0.03	0.33 ± 0.06
1800–2000 s	θ	0.46 ± 0.06	0.49 ± 0.07	0.48 ± 0.09	0.40 ± 0.07
	slow γ	0.43 ± 0.10	0.45 ± 0.09	0.16 ± 0.02	0.40 ± 0.05
	fast γ	0.49 ± 0.09	0.49 ± 0.10	0.12 ± 0.02	0.36 ± 0.05

**Table 5:** The results of MI.

	WT	TMAS + WT	AD	TMAS + AD
θ	0.25 ± 0.07	0.29 ± 0.11	0.15 ± 0.09	0.21 ± 0.09
Slow γ	0.45 ± 0.11	0.46 ± 0.10	0.08 ± 0.03	0.17 ± 0.05
Fast γ	0.51 ± 0.10	0.52 ± 0.11	0.11 ± 0.04	0.16 ± 0.05

**Table 6:** The Modulation Index of the PAC.

	WT	TMAS + WT	AD	TMAS + AD
θ—slow γ (×10^−4^)	5.52 ± 0.86	6.43 ± 0.47	3.10 ± 0.72	4.14 ± 1.00
θ—fast γ (×10^−4^)	4.40 ± 1.35	4.56 ± 1.86	1.86 ± 0.26	2.04 ± 0.76

## Data Availability

The original contributions presented in this study are included in the article. Further inquiries can be directed to the corresponding author.

## Abbreviations

The following abbreviations are used in this manuscript:

AD	Alzheimer’s Disease
EEG	Electroencephalogram
LFP	local Field Potential
MI	Mutual Information
NOR	Novel Object Recognition
PAC	Phase-Amplitude Coupling
TMAS	Transcranial Magneto-Acoustic Stimulation
